# Invasive Pneumococcal Disease and Pandemic (H1N1) 2009, Denver, Colorado, USA

**DOI:** 10.3201/eid1802.110714

**Published:** 2012-02

**Authors:** George E. Nelson, Kenneth A. Gershman, David L. Swerdlow, Bernard W. Beall, Matthew R. Moore

**Affiliations:** Centers for Disease Control and Prevention, Atlanta, Georgia, USA (G.E. Nelson, D.L. Swerdlow, B.W. Beall, M.R. Moore);; Colorado Department of Health and the Environment, Denver, Colorado, USA (K.A. Gershman)

**Keywords:** invasive pneumococcal disease, IPD, streptococci, influenza, bacteria, viruses, pandemic (H1N1) 2009, Denver, Colorado, United States

## Abstract

Pneumococcal prevention strategies should be emphasized during future influenza pandemics.

## Medscape CME Activity

Medscape, LLC is pleased to provide online continuing medical education (CME) for this journal article, allowing clinicians the opportunity to earn CME credit.

This activity has been planned and implemented in accordance with the Essential Areas and policies of the Accreditation Council for Continuing Medical Education through the joint sponsorship of Medscape, LLC and Emerging Infectious Diseases. Medscape, LLC is accredited by the ACCME to provide continuing medical education for physicians.

Medscape, LLC designates this Journal-based CME activity for a maximum of 1 *AMA PRA Category 1 Credit(s)*^TM^. Physicians should claim only the credit commensurate with the extent of their participation in the activity.

All other clinicians completing this activity will be issued a certificate of participation. To participate in this journal CME activity: (1) review the learning objectives and author disclosures; (2) study the education content; (3) take the post-test with a 70% minimum passing score and complete the evaluation at www.medscape.org/journal/eid; (4) view/print certificate.

**Release date: January 25, 2011; Expiration date: January 25, 2012**

## Learning Objectives

Upon completion of this activity, participants will be able to:

- Evaluate the epidemiology of pandemic (H1N1) 2009 in cases of invasive pneumococcal disease (IPD).
- Distinguish variables associated with IPD during the period of circulating pandemic (H1N1) 2009.
- Assess patterns of vaccination and antiviral treatment among patients with IPD.
- Analyze the severity of IPD during the period of circulating pandemic (H1N1) 2009.

## CME Editor

**Thomas J. Gryczan, MS,** Technical Writer/Editor, *Emerging Infectious Diseases. Disclosure: Thomas J. Gryczan, MS, has disclosed no relevant financial relationships.*

## CME Author

**Charles P. Vega, MD,** Health Sciences Clinical Professor; Residency Director, Department of Family Medicine, University of California, Irvine. *Disclosure: Charles P. Vega, MD, has disclosed no relevant financial relationships.*

## Authors

Disclosures: **George E. Nelson, MD; Kenneth A. Gershman, MD, MPH; David L. Swerdlow, MD; Bernard W. Beall, PhD;** and **Matthew R. Moore, MD, MPH, have** disclosed no relevant financial relationships.

## References

[1] Morens DM, Taubenberger JK, Fauci AS. Predominant role of bacterial pneumonia as a cause of death in pandemic influenza: implications for pandemic influenza preparedness. J Infect Dis. 2008;198:962–70. 10.1086/591708 18710327 PMC2599911

[2] Jain S, Kamimoto L, Bramley AM, Schmitz AM, Benoit SR, Louie J, Hospitalized patients with 2009 H1N1 influenza in the United States, April–June 2009. N Engl J Med. 2009;361:1935–44. 10.1056/NEJMoa0906695 19815859

[3] Centers for Disease Control and Prevention (CDC). Hospitalized patients with novel influenza A (H1N1) virus infection—California, April–May, 2009. MMWR Morb Mortal Wkly Rep. 2009;58:536–41. 19478723

[4] Ampofo K, Herbener A, Blaschke AJ, Heyrend C, Poritz M, Korgenski K, Association of 2009 pandemic influenza A (H1N1) infection and increased hospitalization with parapneumonic empyema in children in Utah. Pediatr Infect Dis J. 2010;29:905–9. 10.1097/INF.0b013e3181df2c70 20407400 PMC3153298

[5] Pilishvili T, Lexau C, Farley MM, Hadler J, Harrison LH, Bennett NM, Sustained reductions in invasive pneumococcal disease in the era of conjugate vaccine. J Infect Dis. 2010;201:32–41. 10.1086/648593 19947881

[6] Fine MJ, Auble TE, Yealy DM, Hanusa BH, Weissfeld LA, Singer DE, A prediction rule to identify low-risk patients with community-acquired pneumonia. N Engl J Med. 1997;336:243–50. 10.1056/NEJM199701233360402 8995086

[7] To KK, Chan KH, Li IW, Tsang TY, Tse H, Chan JF, Viral load in patients infected with pandemic H1N1 2009 influenza A virus. J Med Virol. 2010;82:1–7. 10.1002/jmv.21664 19950247 PMC7167040

[8] Witkop CT, Duffy MR, Macias EA, Gibbons TF, Escobar JD, Burwell KN, Novel influenza A (H1N1) outbreak at the US Air Force Academy: epidemiology and viral shedding duration. Am J Prev Med. 2010;38:121–6. 10.1016/j.amepre.2009.10.005 19850440

[9] Väkeväinen M, Eklund C, Eskola J, Käyhty H. Cross-reactivity of antibodies to type 6B and 6A polysaccharides of *Streptococcus pneumoniae*, evoked by pneumococcal conjugate vaccines, in infants. J Infect Dis. 2001;184:789–93. 10.1086/322984 11517443

[10] Carvalho MG, Pimenta FC, Gertz RE Jr, Joshi HH, Trujillo AA, Keys LE, PCR-based quantitation and clonal diversity of the current prevalent invasive serogroup 6 pneumococcal serotype, 6C, in the United States in 1999 and 2006 to 2007. J Clin Microbiol. 2009;47:554–9. 10.1128/JCM.01919-08 19116353 PMC2650953

[11] Centers for Disease Control and Prevention. Update influenza activity—United States, 2009–2010 season. MMWR Morb Mortal Wkly Rep. 2010;59:901–8. 20671661

[12] Walter ND, Taylor TH, Shay DK, Thompson WW, Brammer L, Dowell SF, ; Active Bacterial Core Surveillance Team. Influenza circulation and the burden of invasive pneumococcal pneumonia during a non-pandemic period in the United States. Clin Infect Dis. 2010;50:175–83. 10.1086/649208 20014948

[13] Fiore AE, Uyeki TM, Broder K, Finelli L, Euler GL, Singleton JA, ; Centers for Disease Control and Prevention. Prevention and control of influenza with vaccines. Recommendations of the Advisory Committee on Immunization Practices (ACIP), 2010. MMWR Recomm Rep. 2010;59:1–62. 20689501

[14] McCullers JA, McAuley JL, Browall S, Iverson AR, Boyd KL, Henriques Normark B. Influenza enhances susceptibility to natural acquisition of and disease due to *Streptococcus pneumoniae* in ferrets. J Infect Dis. 2010;202:1287–95. 10.1086/656333 20822454 PMC3249639

[15] Estenssoro E, Ríos FG, Apezteguía C, Reina R, Neira J, Ceraso DH, Pandemic 2009 influenza A in Argentina: a study of 337 patients on mechanical ventilation. Am J Respir Crit Care Med. 2010;182:41–8. 10.1164/201001-0037OC 20203241

[16] Zakikhany K, Degail MA, Lamagni T, Waight P, Guy R, Zhao H, Increase in invasive Streptococcus pyogenes and Streptococcus pneumoniae infections in England, December 2010 to January 2011. Euro Surveill. 2011;16:pii:19785. 10.1164/201001-0037OC 21315057

[17] Euler GL, Lu P, Singleton JA; Centers for Disease Control and Prevention. Vaccination coverage among U.S. adults, National Immunization Survey, Adults, 2007 [cited 2010 Jul 1]. http://www.cdc.gov/vaccines/stats-surv/nis/downloads/nis-adult-summer-2007.pdf

[18] Byington CL, Samore MH, Stoddard GJ, Barlow S, Daly J, Korgenski K, Temporal trends of invasive disease due to *Streptococcus pneumoniae* among children in the intermountain west: emergence of nonvaccine serogroups. Clin Infect Dis. 2005;41:21–9. 10.1086/430604 15937758

[19] Whitney CG, Farley MM, Hadler J, Harrison LH, Bennett NM, Lynfield R, Decline in invasive pneumococcal disease after the introduction of protein-polysaccharide conjugate vaccine. N Engl J Med. 2003;348:1737–46. 10.1056/NEJMoa022823 12724479

[20] Lexau CA, Lynfield R, Danila R, Pilishvili T, Facklam R, Farley MM, Changing epidemiology of invasive pneumococcal disease among older adults in the era of pediatric pneumococcal conjugate vaccine. JAMA. 2005;294:2043–51. 10.1001/jama.294.16.2043 16249418

[21] Moore MR, Gertz RE Jr, Woodbury RL, Barkocy-Gallagher GA, Schaffner W, Lexau C, Population snapshot of emergent *Streptococcus pneumoniae* serotype 19A in the United States, 2005. J Infect Dis. 2008;197:1016–27. 10.1086/528996 18419539

[22] Calbo E, Garau J. Invasive pneumococcal disease in children: changing serotypes and clinical expression of disease. Clin Infect Dis. 2005;41:1821–2. 10.1086/498316 16288412

[23] Hicks LA, Harrison LH, Flannery B, Hadler JL, Schaffner W, Craig AS, Incidence of pneumococcal disease due to non-pneumococcal conjugate vaccine (PCV7) serotypes in the United States during the era of widespread PCV7 vaccination, 1998–2004. J Infect Dis. 2007;196:1346–54. 10.1086/521626 17922399

[24] Centers for Disease Control and Prevention. Updated interim recommendations for the use of antiviral medications in the treatment and prevention of influenza for the 2009–2010 season. Centers for Disease Control and Prevention; updated 2009 Dec 7 [cited 2010 Jul 1]. http://www.cdc.gov/H1N1flu/recommendations.htm

[25] Gray GC, Mitchell BS, Tueller JE, Cross ER, Amundson DE. Pneumonia hospitalizations in the US Navy and Marine Corps: rates and risk factors for 6,522 admissions, 1981–1991. Am J Epidemiol. 1994;139:793–802. 8178792 10.1093/oxfordjournals.aje.a117076

[26] Balicer RD, Zarka S, Levine H, Klement E, Sela T, Porat N, Control of *Streptococcus pneumoniae* serotype 5 epidemic of severe pneumonia among young army recruits by mass antibiotic treatment and vaccination. Vaccine. 2010;28:5591–6. 10.1016/j.vaccine.2010.06.031 20599301 PMC7126119

[27] Berk SL, Gage KA, Holtsclaw-Berk SA, Smith JK. Type 8 pneumococcal pneumonia: an outbreak on an oncology ward. South Med J. 1985;78:159–61. 10.1097/00007611-198502000-00010 3975711

[28] DeMaria A Jr, Browne K, Berk SL, Sherwood EJ, McCabe WR. An outbreak of type 1 pneumococcal pneumonia in a men’s shelter. JAMA. 1980;244:1446–9. 10.1001/jama.1980.03310130024022 7420632

[29] Nuorti JP, Butler JC, Crutcher JM, Guevara R, Welch D, Holder P, An outbreak of multidrug-resistant pneumococcal pneumonia and bacteremia among unvaccinated nursing home residents. N Engl J Med. 1998;338:1861–8. 10.1056/NEJM199806253382601 9637804

[30] Centers for Disease Control and Prevention. Outbreak of pneumococcal pneumonia among unvaccinated residents of a nursing home—New Jersey, April 2001. MMWR Morb Mortal Wkly Rep. 2001;50:707–10. 11787578

[31] Gleich S, Morad Y, Echague R, Miller JR, Kornblum J, Sampson JS, *Streptococcus pneumoniae* serotype 4 outbreak in a home for the aged: report and review of recent outbreaks. Infect Control Hosp Epidemiol. 2000;21:711–7. 10.1086/501717 11089655

[32] Quick RE, Hoge CW, Hamilton DJ, Whitney CJ, Borges M, Kobayashi JM. Underutilization of pneumococcal vaccine in nursing home in Washington State: report of a serotype-specific outbreak and a survey. Am J Med. 1993;94:149–52. 10.1016/0002-9343(93)90176-P 8430710

[33] Cherian T, Steinhoff MC, Harrison LH, Rohn D, McDougal LK, Dick J. A cluster of invasive pneumococcal disease in young children in child care. JAMA. 1994;271:695–7. 10.1001/jama.1994.03510330073037 8309033

[34] Craig AS, Erwin PC, Schaffner W, Elliott JA, Moore WL, Ussery XT, Carriage of multidrug-resistant *Streptococcus pneumoniae* and impact of chemoprophylaxis during an outbreak of meningitis at a day care center. Clin Infect Dis. 1999;29:1257–64. 10.1086/313451 10524972

[35] Rauch AM, O'Ryan M, Van R, Pickering LK. Invasive disease due to multiply resistant *Streptococcus pneumoniae* in a Houston, Tex, day-care center. Am J Dis Child. 1990;144:923–7. 2378341 10.1001/archpedi.1990.02150320087033

[36] Centers for Disease Control. Outbreak of invasive pneumococcal disease in a jail—Texas, 1989. JAMA. 1989;262:3257. 10.1001/jama.1989.03430230027008 2585665

[37] Centers for Disease Control and Prevention (CDC). Outbreak of invasive pneumococcal disease in a jail—Texas, 1989. MMWR Morb Mortal Wkly Rep. 1989;38:733–4. 2509879

[38] Hoge CW, Reichler MR, Dominguez EA, Bremer JC, Mastro TD, Hendricks KA, An epidemic of pneumococcal disease in an overcrowded, inadequately ventilated jail. N Engl J Med. 1994;331:643–8. 10.1056/NEJM199409083311004 8052273

[39] Romney MG, Hull MW, Gustafson R, Sandhu J, Champagne S, Wong T, Large community outbreak of *Streptococcus pneumoniae* serotype 5 invasive infection in an impoverished, urban population. Clin Infect Dis. 2008;47:768–74. 10.1086/591128 18690803

[40] Hurt AC, Baas C, Deng YM, Roberts S, Kelso A, Barr IG. Performance of influenza rapid point-of-care tests in the detection of swine lineage A(H1N1) influenza viruses. Influenza Other Respi Viruses. 2009;3:171–6. 10.1111/j.1750-2659.2009.00086.x 19627374 PMC4634687

