# Supplementary material for: Invasive Pneumococcal Disease and Pandemic (H1N1) 2009, Denver, Colorado, USA
Source: Emerg Infect Dis. 2012 Feb;18(2):208–16. doi: 10.3201/eid1802.110714 (PMC3310448; doi:10.3201/eid1802.110714)
Supplement: Related texts

# Research article: Invasive Pneumococcal Disease and Pandemic (H1N1) 2009, Denver, Colorado, USA

**DOI:** 10.3201/eid1802.110714

**Authors:** George E. Nelson, Kenneth A. Gershman, David L. Swerdlow, Bernard W. Beall, Matthew R. Moore

**Affiliations:** Centers for Disease Control and Prevention, Atlanta, Georgia, USA (G.E. Nelson, D.L. Swerdlow, B.W. Beall, M.R. Moore);; Colorado Department of Health and the Environment, Denver, Colorado, USA (K.A. Gershman)

## Abstract

Pneumococcal pneumonia was a complication during previous influenza pandemics but was not evident initially during pandemic (H1N1) 2009. During October 2009 in Denver, Colorado, USA, invasive pneumococcal disease (IPD) and pandemic (H1N1) 2009 peaked simultaneously, which suggests a link. We compared cases of IPD in October 2009 with cases in February 2009, the most recent peak month of seasonal influenza. During October 2009, we observed 58 IPD cases, which was 3× the average number of IPD cases that usually occur in October in Denver. Patients with IPD in October 2009 were younger and more likely to have chronic lung disease than patients who had IPD in February 2009; a total of 10/47 patients had influenza, and 33/53 patients had influenza-like illness. Thus, ≈17%–62% cases of IPD may have been associated with pandemic (H1N1) 2009. Pneumococcal disease prevention strategies should be emphasized during future influenza pandemics.

Pneumonia caused by *Streptococcus pneumoniae* (pneumococci) was a frequent complication of influenza during previous pandemics. In 1 autopsy series, ≈20% of deaths during the 1918 influenza pandemic were associated with pneumococci (*1*). Pandemic (H1N1) 2009 was the first pandemic in which pneumococcal and influenza vaccines and antiviral drug treatment had the potential to change the interaction between pneumococcal infection and influenza.

Among early cases of pandemic (H1N1) 2009, pneumococcal complications were rarely reported (*2**–**4*). However, in October 2009, the Colorado Department of Public Health and Environment identified a substantial increase in cases of invasive pneumococcal disease (IPD) in the Denver metropolitan area, concurrent with a peak in pandemic (H1N1) 2009–associated hospitalizations, raising the question of the role of this pandemic (H1N1) 2009 virus. We evaluated the IPD cases in October 2009 in terms of age, prevalence of concurrent conditions, severity of illness, evidence of co-infection with pandemic (H1N1) 2009 virus, use of antiviral drugs, and influenza and pneumococcal vaccination. We also assessed the possible contribution of changes in laboratory practices to the increase in reported IPD cases.

## Methods

### Epidemiologic Investigation

Classification as IPD required isolation of *S*. *pneumoniae* from a normally sterile site, such as blood or cerebrospinal fluid. IPD cases were identified through the Active Bacterial Core surveillance (ABCs), a population- and laboratory-based system run continuously since 2000 in the 5-county Denver metropolitan area (population 2.4 million). To evaluate the magnitude of the apparent increase in IPD cases during October 2009, we compared the number of cases during that month to the mean number of cases occurring each October during 2004–2008 because the decrease in IPD rates that followed introduction of the 7-valent pneumococcal conjugate vaccine (PCV7) for US infants stabilized by 2004 (*5*).

To evaluate if IPD cases in October 2009 were epidemiologically different from IPD cases in previous, nonpandemic years, we compared the October 2009 cases to IPD cases in February 2009, the most recent local peak in seasonal influenza (H1N1). If the October 2009 cases were associated with pandemic (H1N1) 2009 and if this influenza affected IPD risk differently than seasonal influenza, we would expect the epidemiology of October 2009 IPD cases to differ from a month with predominant nonpandemic, seasonal influenza (H1N1) circulation. Data analyzed included epidemiologic information from the standard ABCs case report form (www.cdc.gov/abcs/files/ABCs_case_report_form_2009.pdf). More detailed information on initial symptoms, diagnostic testing, clinical laboratory information, and clinical management was collected by chart abstraction.

Chart abstraction included review of physician notes, consultation reports, and laboratory results included in patients’ medical records. For October 2009 cases, supplementary information was obtained from interviews with patients or their surrogates. For comparisons of underlying conditions and basic demographics, we ensured consistent methods across time by comparing data derived only from the ABCs case report form. Most October case-patients had pneumonia; for these, severity of illness was assessed by using the Pneumonia Severity Index, a well-established scoring system that incorporates concurrent conditions, laboratory findings, and vital signs at clinical presentation (*6*). Additional data available for only October 2009 cases included vaccination, antiviral drug treatment, serologic test results, and intensive care unit (ICU) admission.

Influenza-associated hospitalizations were identified by using the Colorado Emerging Infections Program. This program defines laboratory-confirmed influenza infection as any positive rapid test or pandemic (H1N1) 2009 virus–specific real-time PCR result in a hospitalized resident of the surveillance area.

To identify the upper limit of potential pandemic (H1N1) 2009 cases among the October 2009 IPD cases, we used 2 approaches. First, because recent data (*7**,**8*) suggest that the sensitivity of PCR for pandemic (H1N1) 2009 virus decreases 5 days after symptom onset, patients with influenza-like illness (ILI) (fever plus cough or sore throat) and negative or unknown PCR results >5 days before the date of pneumococcal culture were considered to be potentially associated with pandemic (H1N1) 2009. Second, all IPD cases in excess of 2 SD above the mean number of IPD cases in October during 2004–2008 were defined as cases that may have been associated with pandemic (H1N1) 2009. Health care providers and the Colorado Immunization Information System were contacted to verify vaccination status for PCV7, pneumococcal polysaccharide vaccine (PPV23), and seasonal influenza and pandemic (H1N1) 2009 vaccines.

### Laboratory Methods

*S*. *pneumoniae* isolates were serotyped by using type-specific antisera and observation of the Quellung reaction at the Streptococcus Laboratory (Centers for Disease Control and Prevention, Atlanta, GA, USA). For analysis, pneumococcal serotypes were grouped as follows: PCV7 serotypes (4, 6B, 6A, 9V, 14, 18C, 19F, and 23F); PPV23 serotypes (PCV7 serotypes plus 1, 2, 3, 5, 7F, 8, 9N, 10A, 11A, 12F, 15B, 17F, 19A, 20, 22F, and 33F); and all other serotypes and nontypeable pneumococci (not included in either vaccine). Serotype 6A was included in PCV7 serotypes because of cross-reactivity of 6A and 6B (*9*). Serotype 6C strains were identified within serogroup 6 by using PCR (*10*). To determine whether IPD cases were caused by an outbreak of a single pneumococcal strain, we compared serotypes causing IPD in the Denver area in October 2009 with those causing IPD in previous years.

Influenza diagnostic methods investigated were real-time reverse transcriptase PCR, rapid influenza test, direct or indirect fluorescent antibody, serologic analysis, and viral culture. Test results were separated by virus identification (A, B, or both) and influenza A subtype (H1, H3, pandemic [H1N1] 2009, unsubtypeable, or other). We surveyed 13 clinical laboratories serving the 16 reporting hospitals to assess total numbers of IPD cases and total positive blood cultures in September–October 2009 and September–October 2008, and to identify if there had been any changes in laboratory procedures related to blood culturing.

### Statistical Analysis

Data were analyzed with SAS version 9.2 (SAS Institute, Cary, NC, USA). The χ^2^ test was used to compare proportions. Medians were compared by using the Wilcoxon ranked-sum test; p values <0.05 were considered significant.

## Results

### Descriptive Epidemiology

Fifty-eight cases of IPD were identified in the Denver Metropolitan Area during October 2009, which was >3× the October average (mean ± SD 18.4 ± 4.7) during 2004–2008 (Figure). Cases were reported from 16 of 20 Denver area acute care hospitals; these hospitals were distributed throughout the 5-county area. Forty-five cases occurred in February 2009.

**Figure:** Influenza hospitalizations and invasive pneumococcal disease (IPD) cases, Denver, Colorado, USA, 2009–2010 vs. 5-year average (2004–2008). The 2004–2008 average IPD data line is repeated in 2010.

Medical records were abstracted for all 58 cases-patients with IPD (Table 1). Compared with February case-patients, October case-patients were younger (median age 45 years vs. 54 years; p = 0.02), and the proportion of nonelderly case-patients (age <60 years) was higher (45 [78%] of 58 vs. 26 [58%] of 45; p = 0.03). After adjusting for different age distributions of the 2 groups, we found that October 2009 case-patients were more likely to have concurrent conditions, specifically, chronic lung disease (Table 1).

**Table 1:** Epidemiologic characteristics of patients with invasive pneumococcal disease, Denver, Colorado, USA, February and October 2009*

Characteristic	Patients with pandemic (H1N1) 2009, Oct 2009, n = 58	Patients with seasonal influenza, Feb 2009, n = 45	p value
Age, y			
0–<5	6 (10)	0 (0)	NC
5–19	3 (5)	3 (7)	NC
20–39	10 (17)	6 (13)	NC
40–59	26 (45)	17 (38)	NC
>60	13 (22)	19 (42)	NC
Median age (range 2 mo–91 y)	45	54	0.02
Sex			0.120
M	38 (66)	26 (58)	NC
F	20 (34)	16 (36)	NC
Unknown	0	3 (7)	NC
Race†			0.012
White	31 (53)	11 (24)	NC
African American	4 (7)	3 (7)	NC
Asian	0 (0)	2 (4)	NC
Unknown	23 (40)	29 (64)	NC
Ethnicity†			<0.0001
Hispanic	14 (24)	7 (16)	NC
Non-Hispanic	25 (43)	4 (9)	NC
Unknown	19 (33)	34 (76)	NC
Concurrent condition‡			NC
Any	40 (69)	17 (38)	<0.0001
Chronic lung disease	17 (29)	3 (7)	0.0002
Diabetes	5 (9)	6 (13)	0.852
HIV infection	2 (3)	0 (0)	0.247
Liver disease	5 (8.6)	2 (4)	0.276
Smoking	15 (26)	10 (22)	0.669
Hospitalized			
Yes	54 (93)	36 (80)	0.536
No	4 (7)	2(4)	NC
Unknown	0	7 (16)	NC
Tested for influenza§	47 (81)	NA	NC
Outpatient setting	8	NA	NC
Inpatient setting	43	NA	NC
Positive test result for influenza§	10	NA	NC

*Values are no. (%) unless otherwise indicated. NA, not available; NC, not calculated. †Race and ethnicity data required additional patient consent. ‡Underlying concurrent condition comparisons were adjusted for age. §Includes data from supplemental case report form not available for prior years.

### Severity of Illness

Fifty-four (93%) of 58 IPD case-patients were hospitalized, and 47 (81%) had pneumonia. Seven case-patients died (12%), and 19 (33%) were admitted to the ICU. All patients who died were >40 years of age (Table 2). There were no major differences between the October 2009 and February 2009 case-patients in the proportion hospitalized or the case-fatality rate. A high (6/7, 86%) proportion of IPD case-patients 20–39 years of age who were hospitalized in October 2009 were admitted to the ICU. All 42 October pneumonia patients had Pneumonia Severity Index scores >2; the 7 case-patients who died all had pneumonia and scores of 4 or 5, which indicated severe illness and recommended hospitalization.

**Table 2:** Severity of illness for patients with invasive pneumococcal disease, by age group, Denver, Colorado, USA, February and October 2009*

Characteristic	No. (%) patients with pandemic (H1N1) 2009, Oct 2009, n = 58	No. (%) patients with seasonal influenza, Feb 2009, n = 45	p value
Age 0–19 y			
Total	9	3	
Hospitalized†	8 (89)	2 (67)	0.461
Admitted to ICU‡§	1 (13)	NA	NC
Died	0	0	NC
Age 20–39 y			
Total	10	6	
Hospitalized	7 (70)	5 (83)	0.551
Admitted to ICU	6 (86)	NA	NC
Died	0	0	NC
Age 40–59 y			
Total	26	17	
Hospitalized	26 (100)	13 (76)	0.168
Admitted to ICU	8 (31)	NA	NC
Died	3	1	0.532
Age >60 y			
Total	13	19	
Hospitalized	13 (100)	16 (84)¶	NC¶
Admitted to ICU	4 (31)	NA	NC
Died	4	3	0.354
Total			
Hospitalized	54 (93)	36 (80)	0.536
Not hospitalized	4 (7)	2 (4)	
Data missing	0	7 (16)	
Admitted to ICU	19 (33)	NA	NC
Died	7 (12)	4 (9)	0.602

*ICU, intensive care unit; NA, not available; NC, not calculated. †Denominator for percentage of hospitalized cases is total case-patients for each age group. ‡Percentage of those admitted to ICU is percentage of hospitalized case-patients for each age group. §Includes data from supplemental case report form, not available for prior years. ¶Three case-patients with unknown hospitalization information. Of known case-patients, 100% were hospitalized.

### Influenza-associated IPD

Of 58 case-patients with IPD, interviews were completed with 46 (79%). Among the IPD case-patients, 47 (81%) had a documented influenza test during their illness; 4 were evaluated before hospitalization and 43 were tested on or after admission. Nine (19%) were evaluated with rapid tests alone, 8 (17%) with PCR alone, and 26 (55%) with both methods. The type of test used for the remaining 4 case-patients could not be determined. Of 47 case-patients tested for influenza, 10 (21%) had documented influenza virus infection. Therefore, >17% (10/58) of the IPD cases were associated with pandemic (H1N1) 2009.

Of the 10 positive case-patients, 2 were given a diagnosis before admission (1 by PCR and 1 by PCR and rapid test) and 8 were given a diagnosis at admission (all patients had both a rapid test and PCR). For those patients given a diagnosis at admission, only 2 (25%) of the rapid test results were positive and all 8 PCR test results were positive. Of the 7 patients for whom influenza A virus subtype analysis was conducted, 6 had pandemic (H1N1) 2009 virus; the other virus was not subtypeable.

Fifty-three of 58 IPD case-patients had sufficient information to evaluate for preceding ILI. Of these case-patients, 33 (62%) reported symptoms consistent with ILI >5 days before date of the culture that yielded pneumococci. All 10 influenza-positive case-patients reported ILI symptoms. Overall, 31 (94%) of the 33 case-patients with ILI were tested for influenza virus.

On the basis of IPD surveillance in Denver during 2004–2008, the maximum expected number of IPD cases during October of nonpandemic years was 28 (mean ± 2 SD 18 ± 5). If all remaining 30 IPD cases were considered to be excess cases associated with pandemic (H1N1) 2009, then 52% of all cases of IPD in October 2009 may have been associated with pandemic (H1N1) 2009.

### Role of Vaccination

All 9 children had been vaccinated with PCV7 at appropriate ages. Of 38 adults 18–64 years of age, 29 (76%) had indications for PPV23 vaccination and 25 (86%) of them had available vaccination records; of these persons, 3 (12%) were vaccinated. The proportion of persons >65 years of age (universal PPV23 vaccination recommendation) with available vaccination records who were vaccinated was 67% (4/6).

### Antiviral Drug Treatment

Thirty-one (53%) of 58 IPD case-patients received antiviral medications during their illnesses; 20 (61%) of 33 patients who reported ILI symptoms received antiviral drugs and 9 (90%) of the 10 patients who had a positive influenza test result received antiviral drugs. According to medical records, the most common reason for not prescribing antiviral drugs was the duration of time since symptom onset. The 1 patient who had confirmed pandemic (H1N1) 2009–associated IPD who died received antiviral drug treatment 72 hours after admission and 48 hours after confirmatory influenza test results.

### Serotypes of Pneumococci Causing IPD

Among the 47 (81%) cases with available isolates, 75% were caused by serotypes included in PPV23 and 4% by those included in PCV7. Serotype 7F was the predominant serotype identified, and it accounted for 34% of the October cases. Serotype distribution was consistent with overall epidemiology of IPD in Colorado during nonpandemic periods. After adjusting for age, we found that the proportion of all case-patients in Denver with IPD caused by serotype 7F during 2004–2008 increased from 3% to 25%. This increase was observed when analyzing only October (0%–34%; p = 0.025) and all other months (2%–23%; p<0.0001). Among the 9 adult influenza-positive case-patients, 7 of the isolates were serotypes contained in PPV23. Five (71%) of 7 persons with an indication for PPV23 had a serotype covered by PPV23, and only 1 (20%) had received PPV23.

### Laboratory Survey

Among 13 surveyed laboratories, 4 implemented changes in blood culture practices over the preceding year. Two hospitals increased the number of times that blood cultures were evaluated for growth, 1 hospital adopted a different automated culture system, and 1 hospital began using a different skin antiseptic. Eleven laboratories saw a higher number of blood cultures submitted during September–October 2009 than in September–October 2008 (overall increase of 16%). The proportion of cultures positive for pneumococci increased at 9 laboratories by an average of >4 additional isolates, which was a relative increase of 50% over the previous year (Table 3).

**Table 3:** Blood culture practices of 13 laboratories for samples from patients with invasive pneumococcal disease, Denver, Colorado, USA, September–-October 2008 and 2009*

Laboratory ID	Change in procedures	Sep–Oct 2008			Sep–Oct 2009		Change in cultures prepared, no. (%)	% change for SPN+
		No. cultures	No. (%) SPN+		No. cultures	No. (%) SPN+		
1	None	935	3 (0.3)		1,095	3 (0.3)	160 (17)	0
2	None	5,634	11(0.2)		6,515	5 (0.1)	881 (16)	−50
3	None	141	0		297	6 (2)	156 (111)	NC
4	None	2,301	3 (0.1)		2,454	6 (0.2)	153 (7)	100
5	BC on all 3 shifts	2,007	4 (0.2)		2,480	8 (0.3)	473 (24)	50
6	BC on all 3 shifts	1,233	0		1,917	2 (0.1)	684 (55)	NC
7	2009 Feb 1: new blood culture system	2,008	4 (0.2)		2,391	9 (0.4)	383 (19)	100
8	None	2,072	5 (0.2)		2,188	4 (0.2)	116 (6)	0
9	None	1,538	1 (0.1)		1,768	2 (0.1)	230 (15)	0
10	2009 Oct: changed antiseptic	2,423	4 (0.2)		2,407	15 (0.6)	−16 (−1)	200
11	None	820	4 (0.5)		1,079	10 (0.9)	259 (32)	80
12	None	1,456	6 (0.4)		2,178	6 (0.3)	722 (50)	−25
13	None	3,405	5 (0.1)		3,390	9 (0.3)	−15 (0)	200
Total		25,973	50 (0.2)		30,159	85 (0.3)	4,186 (16)	50

*ID, identification; SPN+, positive for *Streptococcus pneumoniae*; NC, the percentage increase from 0 to 2 specimens could not be calculated; BC, blood culture.

## Discussion

An investigation of IPD cases in Denver during October 2009 showed 3× the average number of IPD cases identified during October of the previous 5 years and a notable association with pandemic (H1N1) 2009 virus infection. Our findings do not prove a causal relationship between pandemic (H1N1) 2009 and IPD. However, we confirmed that 10 (17%) of 58 case-patients had influenza, and 2 estimates of the maximum proportion of IPD cases that may have been associated with pandemic (H1N1) 2009 showed that proportion was 52%–62%. Increases in testing for pneumococcal infection were modest and could not account for the magnitude of the increase in IPD incidence that we observed. However, the reported increase by area clinical laboratories in blood culture positivity for pneumococci supports a true increase in IPD incidence.

This investigation highlighted factors that may be distinct to IPD cases associated with pandemic (H1N1) 2009. Previous influenza pandemics have implicated secondary bacterial infection as a complication and cause of serious illness and death (*1*). These studies were based largely on autopsy series and histologic confirmation, but were limited in their ability to evaluate clinical presentation, symptoms, and onset that may be distinct to IPD cases identified during nonpandemic influenza seasons.

During the 2009–10 influenza season, the predominant circulating influenza virus was pandemic (H1N1) 2009 virus (*11*). IPD cases mirrored the epidemiology of pandemic (H1N1) 2009, peaked at the same time, and affected younger persons. Because some cases of IPD occur every October and because some cases of IPD are likely attributable to seasonal influenza (*12*), we compared cases detected during October 2009 with IPD cases seen during a peak month of seasonal influenza activity. Attack rates for pandemic (H1N1) 2009 were likely higher than those for seasonal influenza (H1N1) (*13*). Thus, the October IPD cases were more likely to mirror the epidemiology of pandemic (H1N1) 2009.

Studies in animal models demonstrated that influenza strains during previous pandemics have different abilities to predispose persons to secondary pneumococcal infections (*14*). If pandemic (H1N1) 2009 contributed to the increase in IPD cases during October 2009, we would expect a shift from the baseline IPD epidemiology toward a younger population with conditions known to increase the risk for infection with pandemic (H1N1) 2009 virus. In addition, the prevalence of underlying conditions (especially chronic lung disease) is consistent with a causal association between pandemic (H1N1) 2009 and October 2009 IPD cases.

October 2009 cases were not more severe than February 2009 cases, although our statistical power to identify a significant difference was limited. However, for persons 20–39 years of age, a high proportion of IPD hospital admissions required ICU (there were no prepandemic data for comparison). Data gathered during the pandemic from domestic and international sites (*4**,**15**,**16*) suggested that pandemic (H1N1) 2009–associated IPD was not unique to Denver. However, there were no widespread levels of IPD that were greater than expected. At the time of our investigation in Denver, whether increases in IPD in other ABCs sites were statistically or epidemiologically significant was not clear. Since that time, increases have become apparent in other sites, although these increases were consistently more modest than those observed in Denver and were not as thoroughly investigated. In contrast to the previous 5 years, Denver experienced a peak in IPD in October 2009, which was likely attributable to pandemic (H1N1) 2009, and a second peak in December, which likely represented endemic disease.

Prevention of IPD during future influenza pandemics should focus on vaccination and prompt diagnosis. This influenza pandemic was the first in which pneumococcal vaccines and antiviral drug treatment were available. Among adults with IPD in Denver, vaccination rates for persons 18–64 years of age with indications for vaccination were less than the national rate, and vaccination rates for persons >65 years of age were similar to national estimates (*17*). The increase in IPD cases during October, the peak month of hospitalizations of persons with pandemic (H1N1) 2009, might have been minimized if adults at highest risk for IPD had received the recommended polysaccharide vaccine. Increasing PPV23 coverage in populations with increased risk for IPD is a key prevention measure, especially in anticipation of influenza pandemics.

Introduction of PCV7 into routine childhood immunization programs in the United States resulted in dramatic reductions in rates of pneumococcal-related diseases and major changes in the epidemiology for all age groups (*5**,**18**–**23*). In Denver during October 2009, we identified only 2 cases of IPD caused by serotypes included in PCV7, both in adults.

Whether vaccine against pandemic (H1N1 2009, which became available in Denver during late October, could have reduced the number of pandemic (H1N1) 2009–associated IPD cases is unknown. Antiviral drug administration was sometimes delayed or withheld despite national guidance for treatment even if >48 hours had elapsed from onset of illness (*24*), and such withholding may have changed the clinical course of some of the IPD cases.

Outbreaks of *S*. *pneumoniae* have occurred in many settings (*25**–**39*), and individual serotypes have been implicated in localized outbreaks (*27**–**29**,**31**,**39*). The variety of serotypes identified in this outbreak indicates that the increase in IPD was not attributable to enhanced transmission of a single serotype. To address whether the increase in October 2009 reflected a clonal outbreak of 7F, we analyzed the proportion of IPD cases in Denver that were serotype 7F during 2004–2010. During 2004–2009, the proportion of 7F increased (from 3% to 25%). When we evaluated all months except October during 2004–2009, the proportion of 7F still increased (from 2% to 23%). The proportion of IPD caused by 7F has been increasing in Denver over time and cannot be attributed to an increase in October 2009 alone or the pandemic. Furthermore, the distribution of serotypes was similar to serotype distributions in national (*5*) and Denver-specific IPD cases, which suggested that if pandemic (H1N1) 2009, was causally associated with this outbreak, it facilitated pneumococcal infection without a predilection for any particular serotype.

Our investigation had limitations. Low numbers of cases may have limited our ability to identify differences in the epidemiology of IPD during October 2009 and peak months of seasonal influenza activity. We were also unable to ascertain PPV23 vaccination histories for all cases, which may have underestimated PPV23 use. Of 47 influenza tests ordered, 9 (19%) were only rapid tests. The sensitivity of rapid tests for detecting pandemic (H1N1) 2009 ranged from 20% to 40% (*40*). Twenty-four (41%) of 58 IPD case-patients were not tested for pandemic (H1N1) 2009 by PCR (the standard for detection), which may have underestimated the number of confirmed influenza-associated IPD cases. Some patients with negative test results may have been infected with influenza virus but were tested too late in the course of their illness. Finally, ILI does not capture all influenza cases and cases with influenza within 5 days of pneumococcal culture and not tested samples would not be included for a possible influenza-associated IPD case. In addition, ILI includes symptoms that occur frequently with signs and symptoms of pneumococcal pneumonia and may be a result of the symptom course of IPD rather than preceding influenza infection.

In conclusion, up to two thirds of IPD cases in Denver during October 2009 may have been associated with pandemic (H1N1) 2009. Pandemic influenza may have altered the epidemiology of IPD and shifted the age distribution to younger persons and to persons 18–64 years of age with an increased prevalence of underlying conditions. Missed opportunities for PPV23 vaccination were common. During future influenza pandemics, public health officials should increase awareness of the association between IPD and influenza among persons of greatest risk for influenza-associated IPD. Prevention efforts should include use of pneumococcal vaccines and vaccines for directly preventing influenza infection.

## References

[1] Morens DM, Taubenberger JK, Fauci AS. Predominant role of bacterial pneumonia as a cause of death in pandemic influenza: implications for pandemic influenza preparedness. J Infect Dis. 2008;198:962–70. 10.1086/59170818710327PMC2599911

[2] Jain S, Kamimoto L, Bramley AM, Schmitz AM, Benoit SR, Louie J, Hospitalized patients with 2009 H1N1 influenza in the United States, April–June 2009. N Engl J Med. 2009;361:1935–44. 10.1056/NEJMoa090669519815859

[3] Centers for Disease Control and Prevention (CDC). Hospitalized patients with novel influenza A (H1N1) virus infection—California, April–May, 2009. MMWR Morb Mortal Wkly Rep. 2009;58:536–41.19478723

[4] Ampofo K, Herbener A, Blaschke AJ, Heyrend C, Poritz M, Korgenski K, Association of 2009 pandemic influenza A (H1N1) infection and increased hospitalization with parapneumonic empyema in children in Utah. Pediatr Infect Dis J. 2010;29:905–9. 10.1097/INF.0b013e3181df2c7020407400PMC3153298

[5] Pilishvili T, Lexau C, Farley MM, Hadler J, Harrison LH, Bennett NM, Sustained reductions in invasive pneumococcal disease in the era of conjugate vaccine. J Infect Dis. 2010;201:32–41. 10.1086/64859319947881

[6] Fine MJ, Auble TE, Yealy DM, Hanusa BH, Weissfeld LA, Singer DE, A prediction rule to identify low-risk patients with community-acquired pneumonia. N Engl J Med. 1997;336:243–50. 10.1056/NEJM1997012333604028995086

[7] To KK, Chan KH, Li IW, Tsang TY, Tse H, Chan JF, Viral load in patients infected with pandemic H1N1 2009 influenza A virus. J Med Virol. 2010;82:1–7. 10.1002/jmv.2166419950247PMC7167040

[8] Witkop CT, Duffy MR, Macias EA, Gibbons TF, Escobar JD, Burwell KN, Novel influenza A (H1N1) outbreak at the US Air Force Academy: epidemiology and viral shedding duration. Am J Prev Med. 2010;38:121–6. 10.1016/j.amepre.2009.10.00519850440

[9] Väkeväinen M, Eklund C, Eskola J, Käyhty H. Cross-reactivity of antibodies to type 6B and 6A polysaccharides of *Streptococcus pneumoniae*, evoked by pneumococcal conjugate vaccines, in infants. J Infect Dis. 2001;184:789–93. 10.1086/32298411517443

[10] Carvalho MG, Pimenta FC, Gertz RE Jr, Joshi HH, Trujillo AA, Keys LE, PCR-based quantitation and clonal diversity of the current prevalent invasive serogroup 6 pneumococcal serotype, 6C, in the United States in 1999 and 2006 to 2007. J Clin Microbiol. 2009;47:554–9. 10.1128/JCM.01919-0819116353PMC2650953

[11] Centers for Disease Control and Prevention. Update influenza activity—United States, 2009–2010 season. MMWR Morb Mortal Wkly Rep. 2010;59:901–8.20671661

[12] Walter ND, Taylor TH, Shay DK, Thompson WW, Brammer L, Dowell SF, ; Active Bacterial Core Surveillance Team. Influenza circulation and the burden of invasive pneumococcal pneumonia during a non-pandemic period in the United States. Clin Infect Dis. 2010;50:175–83. 10.1086/64920820014948

[13] Fiore AE, Uyeki TM, Broder K, Finelli L, Euler GL, Singleton JA, ; Centers for Disease Control and Prevention. Prevention and control of influenza with vaccines. Recommendations of the Advisory Committee on Immunization Practices (ACIP), 2010. MMWR Recomm Rep. 2010;59:1–62.20689501

[14] McCullers JA, McAuley JL, Browall S, Iverson AR, Boyd KL, Henriques Normark B. Influenza enhances susceptibility to natural acquisition of and disease due to *Streptococcus pneumoniae* in ferrets. J Infect Dis. 2010;202:1287–95. 10.1086/65633320822454PMC3249639

[15] Estenssoro E, Ríos FG, Apezteguía C, Reina R, Neira J, Ceraso DH, Pandemic 2009 influenza A in Argentina: a study of 337 patients on mechanical ventilation. Am J Respir Crit Care Med. 2010;182:41–8. 10.1164/201001-0037OC20203241

[16] Zakikhany K, Degail MA, Lamagni T, Waight P, Guy R, Zhao H, Increase in invasive Streptococcus pyogenes and Streptococcus pneumoniae infections in England, December 2010 to January 2011. Euro Surveill. 2011;16:pii:19785. 10.1164/201001-0037OC21315057

[17] Euler GL, Lu P, Singleton JA; Centers for Disease Control and Prevention. Vaccination coverage among U.S. adults, National Immunization Survey, Adults, 2007 [cited 2010 Jul 1]. http://www.cdc.gov/vaccines/stats-surv/nis/downloads/nis-adult-summer-2007.pdf

[18] Byington CL, Samore MH, Stoddard GJ, Barlow S, Daly J, Korgenski K, Temporal trends of invasive disease due to *Streptococcus pneumoniae* among children in the intermountain west: emergence of nonvaccine serogroups. Clin Infect Dis. 2005;41:21–9. 10.1086/43060415937758

[19] Whitney CG, Farley MM, Hadler J, Harrison LH, Bennett NM, Lynfield R, Decline in invasive pneumococcal disease after the introduction of protein-polysaccharide conjugate vaccine. N Engl J Med. 2003;348:1737–46. 10.1056/NEJMoa02282312724479

[20] Lexau CA, Lynfield R, Danila R, Pilishvili T, Facklam R, Farley MM, Changing epidemiology of invasive pneumococcal disease among older adults in the era of pediatric pneumococcal conjugate vaccine. JAMA. 2005;294:2043–51. 10.1001/jama.294.16.204316249418

[21] Moore MR, Gertz RE Jr, Woodbury RL, Barkocy-Gallagher GA, Schaffner W, Lexau C, Population snapshot of emergent *Streptococcus pneumoniae* serotype 19A in the United States, 2005. J Infect Dis. 2008;197:1016–27. 10.1086/52899618419539

[22] Calbo E, Garau J. Invasive pneumococcal disease in children: changing serotypes and clinical expression of disease. Clin Infect Dis. 2005;41:1821–2. 10.1086/49831616288412

[23] Hicks LA, Harrison LH, Flannery B, Hadler JL, Schaffner W, Craig AS, Incidence of pneumococcal disease due to non-pneumococcal conjugate vaccine (PCV7) serotypes in the United States during the era of widespread PCV7 vaccination, 1998–2004. J Infect Dis. 2007;196:1346–54. 10.1086/52162617922399

[24] Centers for Disease Control and Prevention. Updated interim recommendations for the use of antiviral medications in the treatment and prevention of influenza for the 2009–2010 season. Centers for Disease Control and Prevention; updated 2009 Dec 7 [cited 2010 Jul 1]. http://www.cdc.gov/H1N1flu/recommendations.htm

[25] Gray GC, Mitchell BS, Tueller JE, Cross ER, Amundson DE. Pneumonia hospitalizations in the US Navy and Marine Corps: rates and risk factors for 6,522 admissions, 1981–1991. Am J Epidemiol. 1994;139:793–802.817879210.1093/oxfordjournals.aje.a117076

[26] Balicer RD, Zarka S, Levine H, Klement E, Sela T, Porat N, Control of *Streptococcus pneumoniae* serotype 5 epidemic of severe pneumonia among young army recruits by mass antibiotic treatment and vaccination. Vaccine. 2010;28:5591–6. 10.1016/j.vaccine.2010.06.03120599301PMC7126119

[27] Berk SL, Gage KA, Holtsclaw-Berk SA, Smith JK. Type 8 pneumococcal pneumonia: an outbreak on an oncology ward. South Med J. 1985;78:159–61. 10.1097/00007611-198502000-000103975711

[28] DeMaria A Jr, Browne K, Berk SL, Sherwood EJ, McCabe WR. An outbreak of type 1 pneumococcal pneumonia in a men’s shelter. JAMA. 1980;244:1446–9. 10.1001/jama.1980.033101300240227420632

[29] Nuorti JP, Butler JC, Crutcher JM, Guevara R, Welch D, Holder P, An outbreak of multidrug-resistant pneumococcal pneumonia and bacteremia among unvaccinated nursing home residents. N Engl J Med. 1998;338:1861–8. 10.1056/NEJM1998062533826019637804

[30] Centers for Disease Control and Prevention. Outbreak of pneumococcal pneumonia among unvaccinated residents of a nursing home—New Jersey, April 2001. MMWR Morb Mortal Wkly Rep. 2001;50:707–10.11787578

[31] Gleich S, Morad Y, Echague R, Miller JR, Kornblum J, Sampson JS, *Streptococcus pneumoniae* serotype 4 outbreak in a home for the aged: report and review of recent outbreaks. Infect Control Hosp Epidemiol. 2000;21:711–7. 10.1086/50171711089655

[32] Quick RE, Hoge CW, Hamilton DJ, Whitney CJ, Borges M, Kobayashi JM. Underutilization of pneumococcal vaccine in nursing home in Washington State: report of a serotype-specific outbreak and a survey. Am J Med. 1993;94:149–52. 10.1016/0002-9343(93)90176-P8430710

[33] Cherian T, Steinhoff MC, Harrison LH, Rohn D, McDougal LK, Dick J. A cluster of invasive pneumococcal disease in young children in child care. JAMA. 1994;271:695–7. 10.1001/jama.1994.035103300730378309033

[34] Craig AS, Erwin PC, Schaffner W, Elliott JA, Moore WL, Ussery XT, Carriage of multidrug-resistant *Streptococcus pneumoniae* and impact of chemoprophylaxis during an outbreak of meningitis at a day care center. Clin Infect Dis. 1999;29:1257–64. 10.1086/31345110524972

[35] Rauch AM, O'Ryan M, Van R, Pickering LK. Invasive disease due to multiply resistant *Streptococcus pneumoniae* in a Houston, Tex, day-care center. Am J Dis Child. 1990;144:923–7.237834110.1001/archpedi.1990.02150320087033

[36] Centers for Disease Control. Outbreak of invasive pneumococcal disease in a jail—Texas, 1989. JAMA. 1989;262:3257. 10.1001/jama.1989.034302300270082585665

[37] Centers for Disease Control and Prevention (CDC). Outbreak of invasive pneumococcal disease in a jail—Texas, 1989. MMWR Morb Mortal Wkly Rep. 1989;38:733–4.2509879

[38] Hoge CW, Reichler MR, Dominguez EA, Bremer JC, Mastro TD, Hendricks KA, An epidemic of pneumococcal disease in an overcrowded, inadequately ventilated jail. N Engl J Med. 1994;331:643–8. 10.1056/NEJM1994090833110048052273

[39] Romney MG, Hull MW, Gustafson R, Sandhu J, Champagne S, Wong T, Large community outbreak of *Streptococcus pneumoniae* serotype 5 invasive infection in an impoverished, urban population. Clin Infect Dis. 2008;47:768–74. 10.1086/59112818690803

[40] Hurt AC, Baas C, Deng YM, Roberts S, Kelso A, Barr IG. Performance of influenza rapid point-of-care tests in the detection of swine lineage A(H1N1) influenza viruses. Influenza Other Respi Viruses. 2009;3:171–6. 10.1111/j.1750-2659.2009.00086.x19627374PMC4634687

# Research article: Invasive Pneumococcal Disease and Pandemic (H1N1) 2009, Denver, Colorado, USA

**DOI:** 10.3201/eid1802.110714

**Authors:** George E. Nelson, Kenneth A. Gershman, David L. Swerdlow, Bernard W. Beall, Matthew R. Moore

**Affiliations:** Centers for Disease Control and Prevention, Atlanta, Georgia, USA (G.E. Nelson, D.L. Swerdlow, B.W. Beall, M.R. Moore);; Colorado Department of Health and the Environment, Denver, Colorado, USA (K.A. Gershman)

## Earning CME Credit

To obtain credit, you should first read the journal article. After reading the article, you should be able to answer the following, related, multiple-choice questions. To complete the questions (with a minimum 70% passing score) and earn continuing medical education (CME) credit, please go to www.medscape.org/journal/eid. Credit cannot be obtained for tests completed on paper, although you may use the worksheet below to keep a record of your answers. You must be a registered user on Medscape.org. If you are not registered on Medscape.org, please click on the New Users: Free Registration link on the left hand side of the website to register. Only one answer is correct for each question. Once you successfully answer all post-test questions you will be able to view and/or print your certificate. For questions regarding the content of this activity, contact the accredited provider, CME@medscape.net. For technical assistance, contact CME@webmd.net. American Medical Association’s Physician’s Recognition Award (AMA PRA) credits are accepted in the US as evidence of participation in CME activities. For further information on this award, please refer to http://www.ama-assn.org/ama/pub/category/2922.html. The AMA has determined that physicians not licensed in the US who participate in this CME activity are eligible for *AMA PRA Category 1 Credits*™. Through agreements that the AMA has made with agencies in some countries, AMA PRA credit may be acceptable as evidence of participation in CME activities. If you are not licensed in the US, please complete the questions online, print the certificate and present it to your national medical association for review.

## Article Title: Invasive Pneumococcal Disease and Pandemic (H1N1) 2009, Denver, Colorado, USA

## CME Questions

**1. You are admitting a 55-year-old woman with a history of chronic obstructive pulmonary disease, type 2 diabetes mellitus, and steatohepatitis. She has had fever and cough for 8 days, and it has grown worse in the last 2 days.You consider whether this patient has influenza, invasive pneumococcal disease (IPD), or both. Which of the following statements regarding the relationship between pandemic (H1N1) 2009 and IPD in the current study is most accurate?**A. Fewer than 20% of cases of IPD featured positive testing for pandemic (H1N1) 2009B. Most cases of IPD did not feature influenza-like illness (ILI)C. More patients had positive testing for pandemic (H1N1) 2009 than for ILID. The sensitivity of rapid influenza testing approached 100%**2. Which of the following variables most characterized pandemic (H1N1) 2009–associated cases of IPD vs IPD cases during the nonpandemic period?**A. Younger age and black raceB. Younger age and higher rates of chronic obstructive pulmonary diseaseC. Female sex and higher rates of diabetesD. Older age and higher rates of liver disease**3. As you take this patient's history, what information from the current study should you consider regarding the use of pneumococcal vaccine and treatment of influenza?**A. Nearly all eligible patients had received the pneumococcal polysaccharide vaccine (PPV23) vaccineB. All children had received the 7-valent pneumococcal conjugate (PCV7) vaccineC. Fewer than 10% of patients received antiviral medicationsD. The most common reason for not prescribing antiviral medications was fear of side effects**4. The patient is diagnosed with influenza (H1N1) and IPD. What was the difference in the severity of cases of IPD in the pandemic vs the nonpandemic periods in the current study?**A. Pandemic (H1N1) 2009 was associated with higher rates of hospitalization and mortalityB. Pandemic (H1N1) 2009 was associated with higher rates of hospitalization onlyC. Pandemic (H1N1) 2009 was associated with higher rates of mortality onlyD. Pandemic (H1N1) 2009 was not associated with higher rates of hospitalization or mortality

## Activity Evaluation

**1:** The activity supported the learning objectives.

Strongly Disagree				Strongly Agree
1	2	3	4	5
**2. The material was organized clearly for learning to occur.**				
Strongly Disagree				Strongly Agree
1	2	3	4	5
**3. The content learned from this activity will impact my practice.**				
Strongly Disagree				Strongly Agree
1	2	3	4	5
**4. The activity was presented objectively and free of commercial bias.**				
Strongly Disagree				Strongly Agree
1	2	3	4	5
